# miR-96 suppresses renal cell carcinoma invasion via downregulation of Ezrin expression

**DOI:** 10.1186/s13046-015-0224-8

**Published:** 2015-09-29

**Authors:** Nengwang Yu, Shuai Fu, Yubao Liu, Zhonghua Xu, Yi Liu, Junwen Hao, Baocheng Wang, Aimin Zhang

**Affiliations:** Urology Department, General Hospital of Jinan Military Command, 25 Shifan Road, Jinan, Shandong 250031 China; Shandong Cancer Hospital & Institute, Shandong Academy of Medical Sciences, 440 Jiyan Road, Jinan, 250117 China; Department of urology, Beijing Tsinghua Changgung Hospital Medical Center, Tsinghua University, 168 Litang Road, Dongxiaokou Town, Changqing District, Beijing, 102218 China; Urology Department, Qilu Hospital, 107 West Wenhua Road, Jinan, Shandong 250012 China; Oncology Department, General Hospital of Jinan Military Command, 25 Shifan Road, Jinan, Shandong 250031 China

**Keywords:** MicroRNA, Renal cancer, Ezrin, Metastasis

## Abstract

**Background:**

The present study examined the role of microRNA (miR)-96 in renal cell carcinoma (RCC) invasion.

**Methods:**

The expression of miR-96 was detected by quantitative reverse transcription-polymerase chain reaction in human RCC cell lines with high (Caki-1) and low (786-O) metastatic potential. Invasive ability and Ezrin expression were assessed in Caki-1 and 786-O cells transfected with a miR-96 mimic or inhibitor using wound healing assays, Transwell assays and western blotting. Expression of miR-96 and Ezrin was also examined in primary RCC samples from 17 patients with metastatic disease and 46 patients who maintained remission during a follow-up period of 37 months.

**Results:**

miR-96 expression was significantly lower in Caki-1compared to786-O cells. The invasive ability of Caki-1 and 786-O cells increased following transfection of cells with miR-96 inhibitor, whereas it decreased following transfection with miR-96 mimic. Ezrin levels were negatively correlated with miR-96 in RCC, and inhibition of Ezrin expression suppressed the miR-96-induced change in invasive ability. The negative correlation between miR-96 and metastasis/Ezrin expression was also observed in human RCC specimens.

**Conclusions:**

These results suggest that miR-96 suppresses RCC invasion by modulating Ezrin expression.

## Background

Despite improvements in available treatments, the prognosis remains poor for locally advanced and metastatic renal cell carcinoma (RCC) [1]. Thus, it is critical to identify the molecules controlling the invasive and metastatic potential of RCC, which may provide new targets for intervention.

MicroRNAs (miRNAs) are a class of small non-coding RNAs that play a key role in cancer progression [2]. miRNAs regulate the expression of many invasion and metastasis-related genes in various types of malignancies [3–5]. Among miRNAs, microRNA-96 (miR-96) was recently shown to be involved in the invasive and metastatic potential of hepatocellular carcinoma [6, 7], breast cancer [8, 9], lung cancer [10],pancreatic cancer [11] and bladder cancer [12]. To the best of our knowledge, the relationship between miR-96 and RCC invasion or metastasis has not been investigated. In the present study, we explored the role of miR-96 in RCC invasion and metastasis.

The expression of miR-96 was detected in RCC cell lines and clinical samples with different metastatic potential. Our data indicated that miR-96 expression was negatively correlated with the metastatic potential of RCC, and overexpression of miR-96 by transfection with synthetic miR-96 oligonucleotides decreased the invasive potential of RCC cells.

Ezrin has been recognized as a molecular linker between the actin cytoskeleton and plasma membrane, and is involved in the maintenance of cell adhesion and cell movement. Increasing evidence has shown that Ezrin is associated with metastasis of various human malignancies [13–22]. The *in silico* analysis of Ezrin and miRNAs using three prediction programs, TargetScan, miRanda and PicTar, revealed that Ezrin is a target of miR-96. We hypothesized that miR-96 may suppress RCC cell invasion via regulation of Ezrin expression and verified this hypothesis in the present study. Ezrin level was shown to be negatively correlated with miR-96 in RCC cell lines, and inhibition of Ezrin expression suppressed the miR-96-induced change in invasive ability. The negative correlation between miR-96 and metastasis/Ezrin expression was also observed in human RCC specimens. These results suggest that miR-96 may suppress RCC invasion through the modulation of Ezrin expression.

## Methods

### Cell culture

Caki-1 and 786-O, which are human RCC cell lines with high and low metastatic potential, respectively, were purchased from the Type Culture Collection of the Chinese Academy of Sciences (Shanghai, China). Caki-1 cells were cultured in McCoy’s 5A medium (Gibco, Grand Island, NY, U.S.) supplemented with 15 % fetal bovine serum (FBS; Shanghai Sangon Biological Engineering Technology and Services Co., Ltd., Shanghai, China), and 786-O cells were cultured in RPMI 1640 (Wisent, Saint-Jean-Baptiste, Canada) supplemented with 10 % FBS.

### Clinical sample collection

Human kidney specimens were obtained from 63 patients who underwent radical nephrectomy for localized clear cell RCC at the General Hospital of Jinan Military Command in China between 2008 and 2013. The collection and use of the samples were reviewed and approved by the Institutional Ethics Committee of General Hospital of Jinan Military Command, and expedited pathological diagnosis and staging of these specimens were performed prior to sampling and transporting them for research. Histological diagnosis was established according to the guidelines of the World Health Organization [23]. Cases were selected according to tissue availability and were not stratified for any known preoperative or pathological prognostic factor. Clinical follow-up data was available for all patients. The median follow-up period for all cases was 37 months (range, 7–65 months). Under the supervision of an experienced pathologist, 63 renal cancer tissue samples were collected (before any treatment was begun) from surgically resected kidneys and immediately stored in liquid nitrogen until RNA or protein extraction.

### Quantitative reverse transcription-polymerase chain reaction (qRT-PCR)

Total RNA was extracted from cells using TRIzol reagent (Invitrogen Life Technologies, Carlsbad, CA, U.S.) according to the manufacturer’s protocol. The expression of miR-96 was measured using the Hairpin-it™ miRNAs qPCR Quantitation Kit (GenePharma, Shanghai, China) with the following primers: Sense 5′-TTTGGCACTAGC ACAT-3′; antisense 5′-GAGCAGGCTGGAGAA-3′. The miRNA synthetic standard in the kit was used as a positive control, according to the manufacturer’s instructions. U6 small nuclear RNA was used as an internal control, with the following primers: Sense 5′-ATTGGAACGATACAGAGAAGAT-3′; antisense 5′-GGAACGCTTCACGAATTT-3′ (GenePharma, Shanghai, China). The relative expression of miR-96 in tissues and cell lines were calculated by the 2^-Δct^ method.

### Transfection

Caki-1 and 786-Ocells were transiently transfected with miR-96 inhibitor, miR-96 mimic and miR-control RNA using Lipofectamine 2000 (Invitrogen). Inhibitor of miR-96 (sequence: 5′-GCAAAAAUGUGCUAGUGCCAAA-3′), mimic of miR-96 (sequence: 5′-UUUGGCACUAGCACAUUUUUGC-3′) and negative miR-control (sequence: 5′-CAGUACUUUUGUGUAGUACAA-3′) were purchased from GenePharma. The negative miR-control sequence was non-homologous to any human genomic sequence in order to eliminate potential non-sequence-specific effects as previously reported [24]. RCC cells were seeded in six-well plates and transfected with 4 nM of miR-96 inhibitor, miR-96 mimic or miR-control.

### Wound healing assay

Caki-1 and 786-O cells transfected with miR-96 mimic, inhibitor or miR-control were cultured as monolayers, synchronized by starving the cells for 24 h in Dulbecco’s Modified Eagle Medium (Gibco) containing 0.1 % FBS, and wounded by removing a wide strip (approximately 300 μm) of cells across the well with a standard 200 μl pipette tip. Wounded monolayers were washed twice to remove nonadherent cells. Wound healing was monitored by phase-contrast microscopy after 24 h culture in 1 % FBS, quantified using Image J software and expressed as the mean percentage of the remaining cell-free area compared with the area of the initial wound.

### Transwell assay

The invasive ability of Caki-1 and 786-O cells transfected with miR-96 mimic, inhibitor or miR-control was determined using Matrigel (BD Pharmingen, San Diego, CA, U.S.)-coated 24-well Transwell chambers, with upper and lower culture compartments separated by polycarbonate membranes with 8-μm pores (Corning, Costar, New York City, U.S.). The bottom chamber was filled with RPMI-1640 medium containing 10 % FBS for 786-Ocells or McCoy’s5A medium with 15 % FBS for Caki-1 cells. The transfected cells (1 × 10^5^) were seeded on the top chamber and incubated at 37 °C with 5 % CO_2_. After 40 h, the cells were removed from the upper surface by scrubbing with a cotton swab, and cells that had migrated to the underside of the membrane were stained with Giemsa (Sigma-Aldrich Co., St. Louis, MO, U.S.). A total of five high-power fields were counted by two independent blinded pathologists, and the mean number of cells per field was calculated. The experiments were performed in triplicate.

To further investigate the role of Ezrin in the inhibition of invasion by miR-96, Caki-1 and 786-O cells were pretreated for 30 min with 10 μM NSC668394, a small molecule inhibitor of Ezrin [25], followed by transfection with miR-control or miR-96 inhibitor for 24 h. Invasive ability was then examined by Transwell assay as described above.

### Western blot analysis

Ezrin expression was assessed by western blotting using standard protocols. Briefly, equal amounts of extracted protein, as determined by Bradford protein assay (Bio-Rad, Hercules, CA, U.S.), were separated by sodium dodecyl sulfate (SDS)-8 % polyacrylamide gel electrophoresis and blotted onto polyvinylidene difluoride membranes (GE Healthcare, Little Chalfont, England). Membranes were probed with Ezrin antibody (Abcam, Cambridge, MA, U.S.). After incubation with peroxidase-coupled secondary antibodies (Abcam), blots were developed using enhanced chemiluminescence reagents and exposed to X-ray films to detect labeled proteins. Membranes were then stripped with Re-Blot Plus (Millipore, Billerica, MA, U.S.) and subsequently reprobed for glyceraldehyde-3-phosphate dehydrogenase (GAPDH; Abcam) as a loading control.

### Data analysis

Negative and positive controls were routinely incorporated for quality control in all the above assays. All analyses were performed using SPSS 13 software. Repeated-measures analysis of variance (ANOVA) tests were used to compare multiple groups and *p* values <0.05 were considered statistically significant. Results are expressed as mean ± standard deviation.

## Results

### Expression of miR-96 in RCC cell lines with varying metastatic potential

**Fig. 1:**
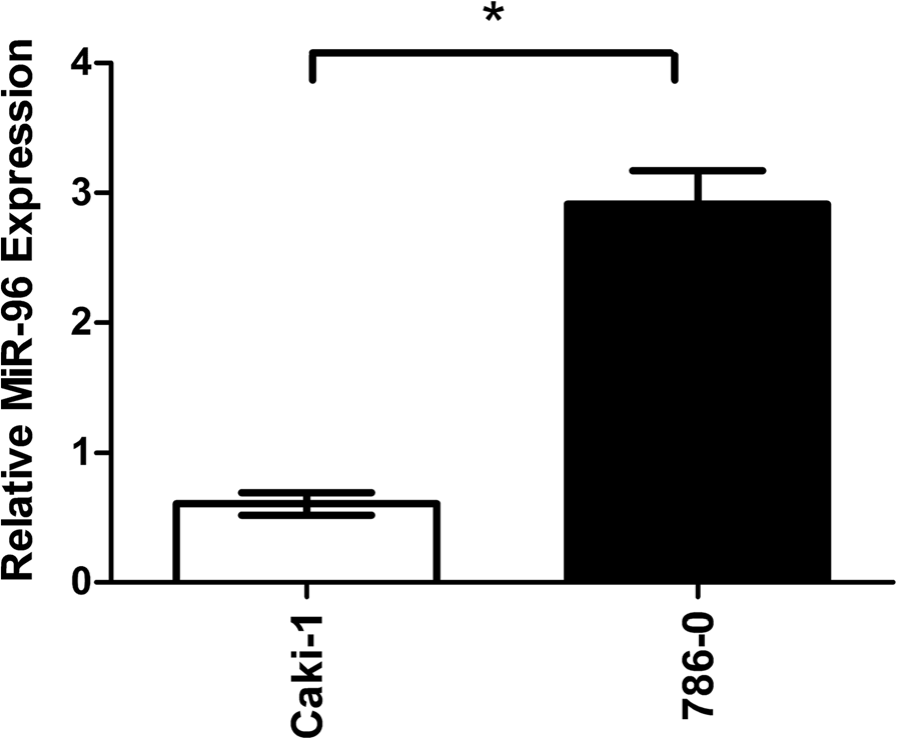
miR-96 expression in renal cell carcinoma (RCC) cell lines with varying metastatic potential. miR-96 expression was detected by quantitative reverse transcription-polymerase chain reaction (RT-PCR). U6 small nuclear RNA was used as an internal control (*p < 0.05)

To evaluate the relationship between miR-96 expression and metastatic potential, miR-96 levels were measured in RCC cell lines with varying metastatic potential by qRT-PCR. The results showed that miR-96 was expressed at a low level in Caki-1 cells with high metastatic potential and at a high level in 786-O cells with low metastatic potential (0.6 ± 0.08 vs. 2.9 ± 0.26, respectively; *P* < 0.05; Fig. 1). These findings provide evidence that miR-96 expression is correlated with the metastatic phenotype of RCC cells.

### Impact of miR-96 expression on *in vitro* invasion by RCC cells

**Fig. 2:**
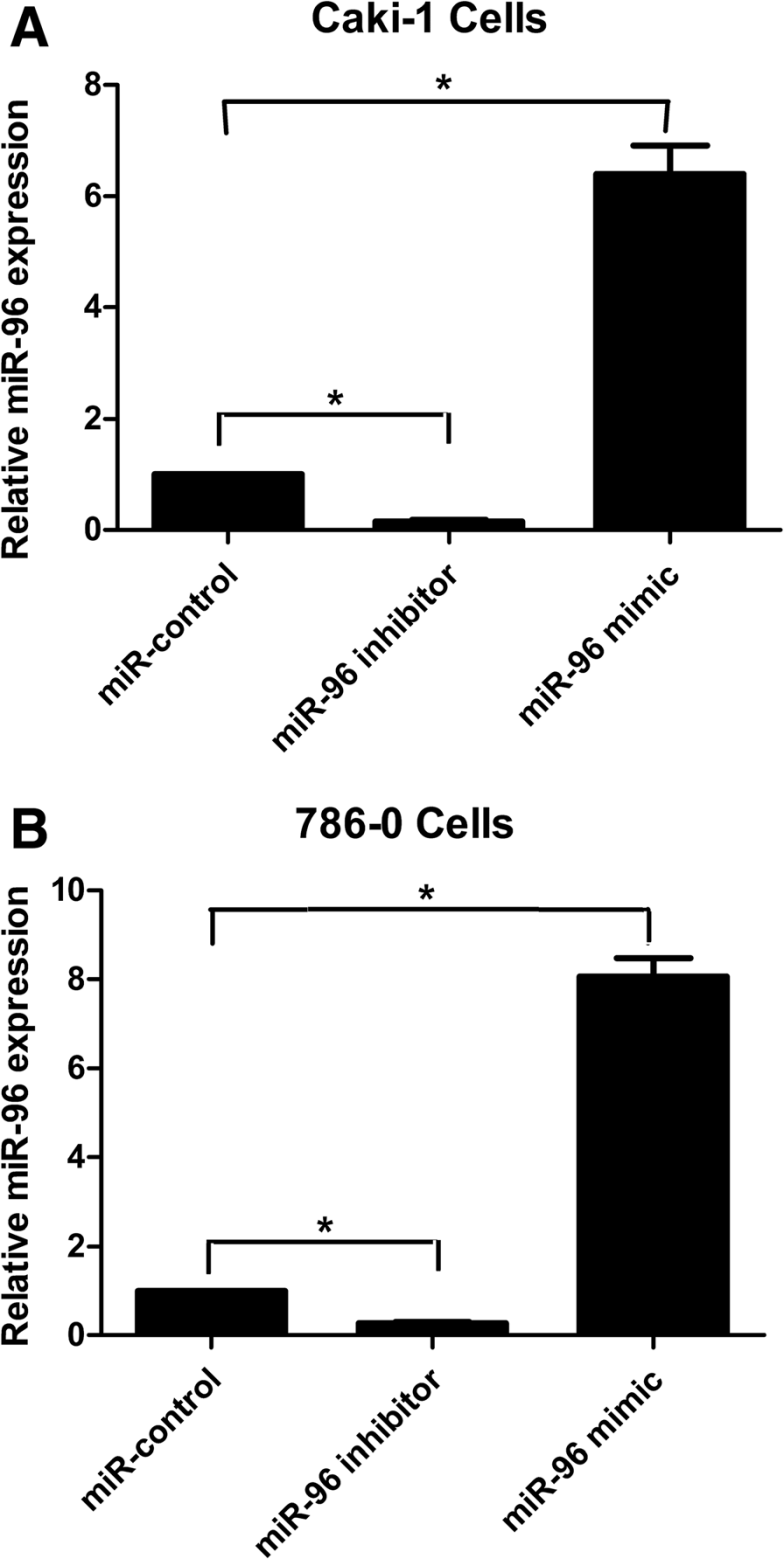
miR-96 expression changes in Caki-1(**a**) and 786-O (**b**) cells following transfection with miR-96 inhibitor or miR-96 mimic compared to miR-control-transfected cells. miR-96 expression in RCC cell lines was detected by quantitative RT-PCR after transfection with the indicated constructs for 24 h (*p < 0.05)

As shown in Fig. 2, miR-96 expression was significantly down- or upregulated in Caki-1 and 786-O cells transfected with the miR-96 inhibitor or miR-96 mimic, respectively, compared to miR-control-transfected cells.

**Fig. 3:**
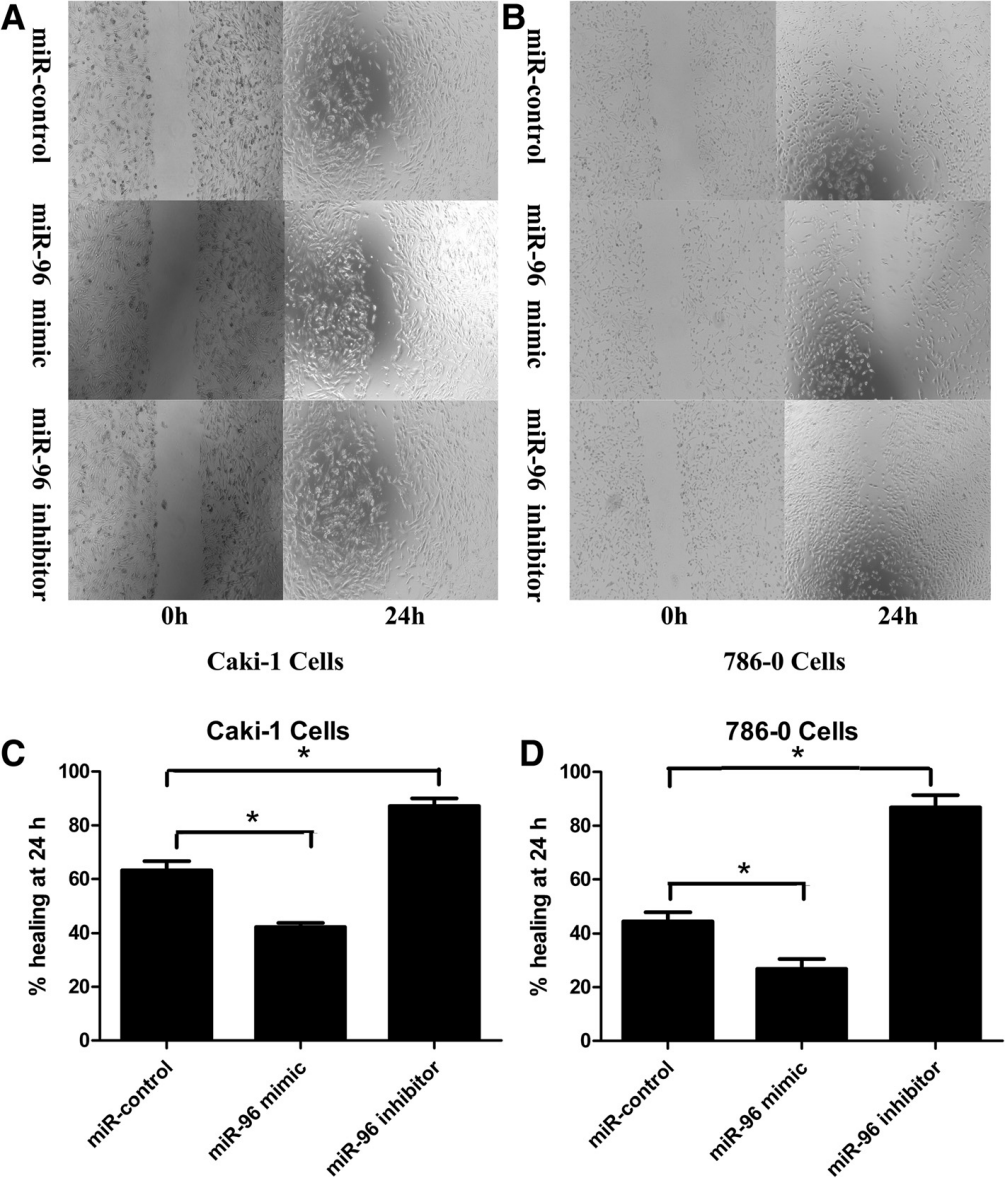
Migration of Caki-1 and 786-O cells transfected with miR-96 inhibitor or miR-96 mimic compared to miR-control-transfected cells. Wound healing assay of Caki-1 cells (**a**) and 786-O cells (**b**) transfected with miR-96 inhibitor, miR-96 mimic or miR-control for 24 h. Percent healing at 24 h was calculated as the mean percentage of the remaining cell-free area compared with the area of the initial wound and showed that migration of Caki-1 (**c**) and 786-O (**d**) cells increased following transfection with miR-96 inhibitor, and decreased following transfection with miR-96 mimic, compared to cells transfected with miR-control (*p < 0.05)

To evaluate the effect of changes in the levels of miR-96 on the invasive ability of RCC cells *in vitro*, functional assays were performed. Cell migration was assessed by wound healing assay, which showed that Caki-1 and 786-O cells transfected with the miR-96 mimic migrated at a slower rate (Caki-1: 42.2 % ± 2.7 % vs. 63.3 % ± 5.87 %, *P* < 0.05; 786-O: 26.9 % ± 6.35 % vs. 44.4 % ± 5.88 %, *P* < 0.05; Fig. 3), whereas cells transfected with miR-96 inhibitor migrated at a faster rate (Caki-1: 87.2 % ± 4.81 % vs. 63.3 % ± 5.87 %, *P* < 0.05; 786-O: 86.8 % ± 7.99 % vs. 44.4 % ± 5.88 %, *P* < 0.05; Fig. 3), compared to cells transfected with negative miR-control.

**Fig. 4:**
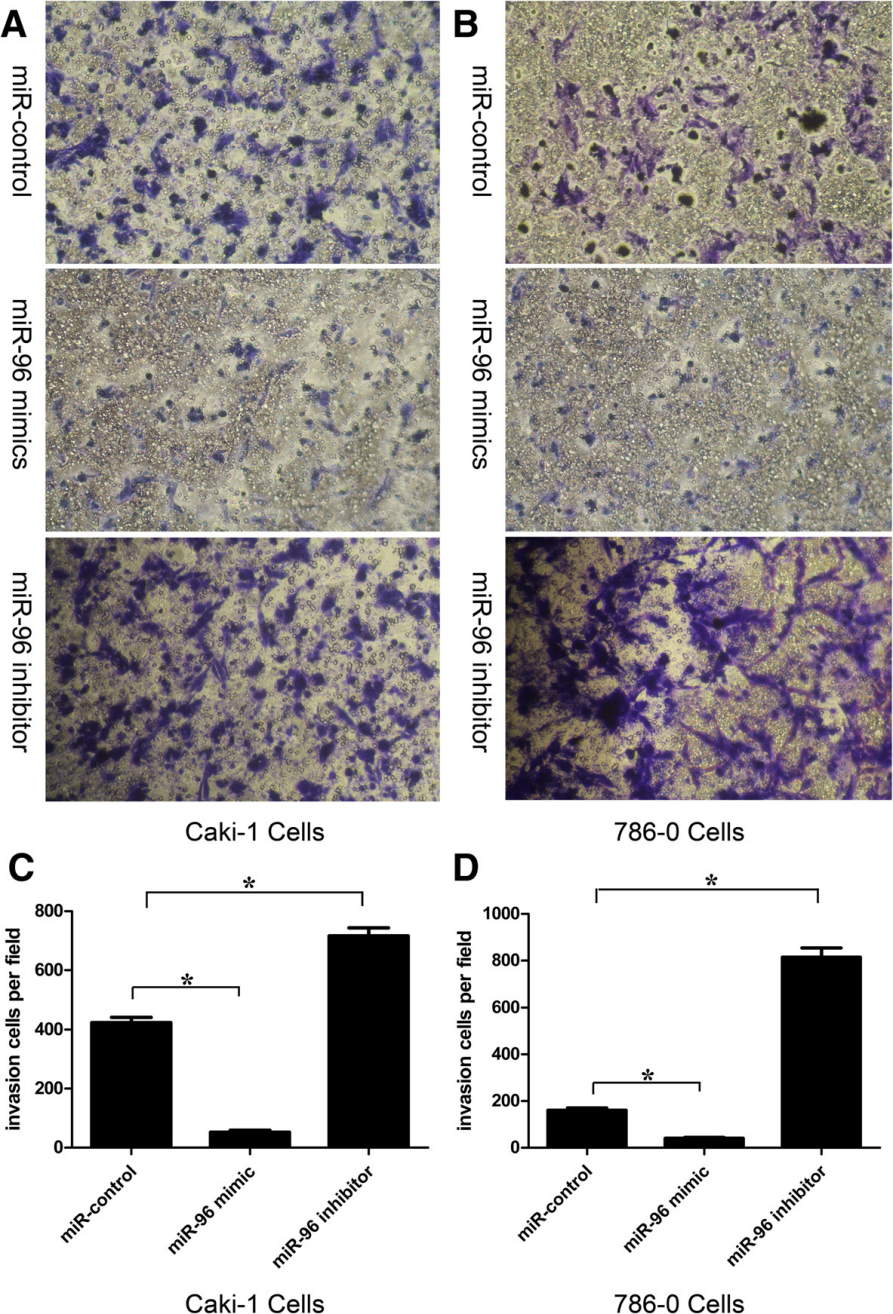
Invasive ability of Caki-1 and 786-O cells transfected with miR-96 inhibitor or miR-96 mimic. Caki-1 cells (**a**) and 786-O cells (**b**) were transfected with miR-96 inhibitor, miR-96 mimic or miR-control for 24 h and invasion was examined by Transwell assay. Invasive ability calculated as the mean number of invading cells per field was increased in Caki-1 (**c**) and 786-O (**d**) cells following transfection with miR-96 inhibitor and decreased following transfection with miR-96 mimic, compared to cells transfected with miR-control (*p < 0.05)

The impact of miR-96 on the invasive ability of RCC cells was assessed by Transwell assay. As shown in Fig. 4, the number of Caki-1 and 786-O cells that invaded through the Matrigel-coated membrane was significantly higher for cells transfected with miR-96 inhibitor(Caki-1:716 ± 62 vs. 424 ± 40, *P* < 0.05; 786-O: 815 ± 88.7 vs. 160 ± 23.1, *P* <0.05, but was significantly lower for cells transfected with miR-96 mimic (Caki-1:52.2 ± 14.9 vs. 424 ± 40, *P* < 0.05; 786-O: 40.6 ± 9.71 vs. 160 ± 23.1, *P* < 0.05), compared to cells transfected with negative miR-control.

### MiR-96 suppresses RCC cell invasion by modulating Ezrin expression

**Fig. 5:**
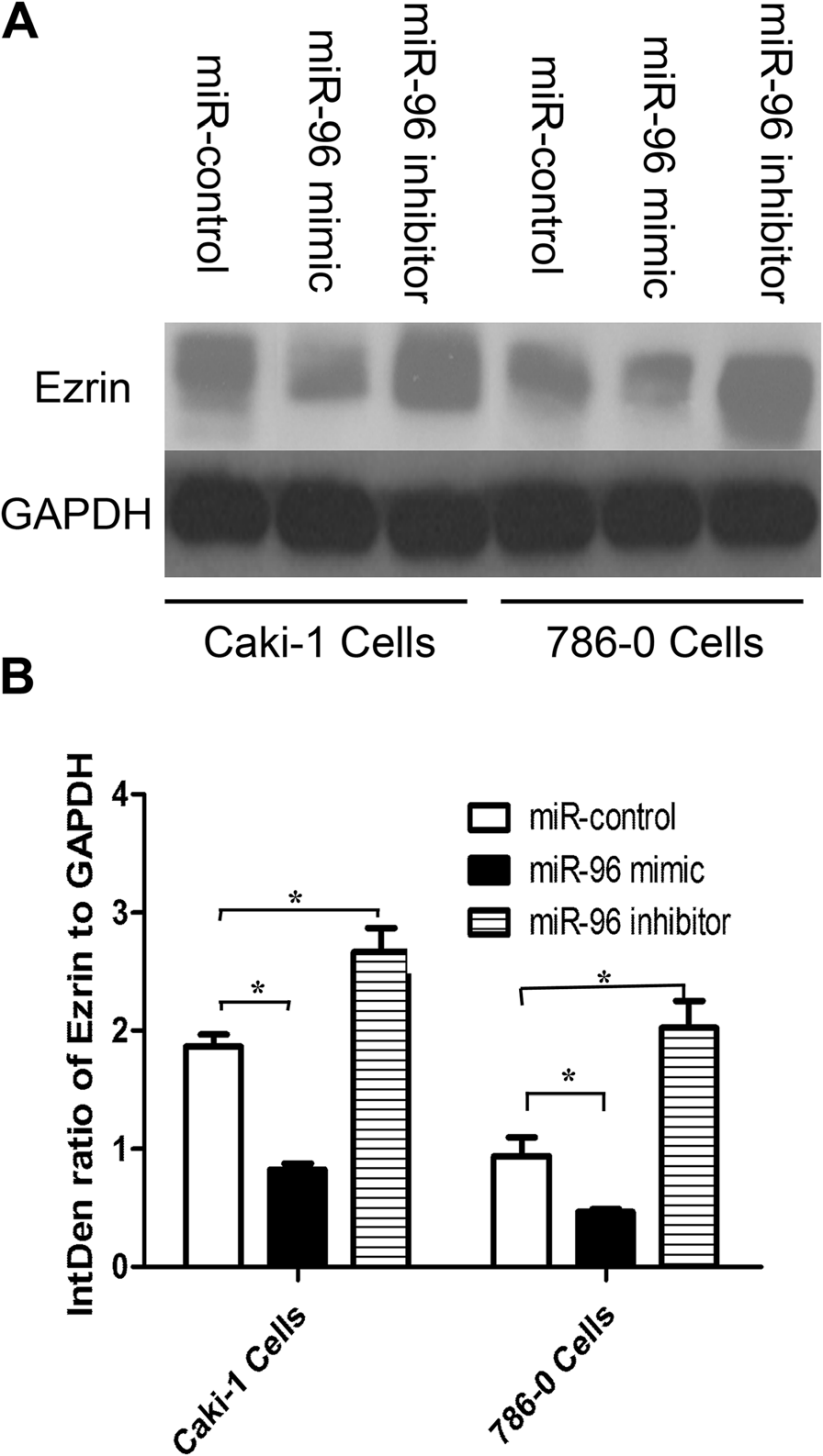
Expression of Ezrin in Caki-1 and 786-O cells transfected with miR-96 mimic, miR-96 inhibitor or miR-control. **a** Ezrin was detected by western blotting using GAPDH as a loading control. **b** Ezrin expression increased following transfection of cells with miR-96 inhibitor, and decreased following transfection with miR-96 mimic, compared to cells transfected with miR-control. IntDen ratio represents the integrated density ratio of Ezrin to GAPDH as quantified by Image J (*p < 0.05)

Ezrin expression was detected by western blotting in Caki-1 and 786-O cells transfected with miR-96 mimic, miR-96 inhibitor or miR-control. As shown in Fig. 5, Ezrin was significantly downregulated in Caki-1 and 786-O cells transfected with miR-96 mimic(Caki-1:0.83 ± 0.08 vs. 1.87 ± 0.18, *P* < 0.05; 786-O: 0.47 ± 0.04 vs.0.94 ± 0.27, *P* < 0.05, and was significantly upregulated in cells transfected with miR-96 inhibitor(Caki-1:2.67 ± 0.35 vs. 1.87 ± 0.18, *P* < 0.05; 786-O: 2.02 ± 0.39 vs.0.94 ± 0.27, *P <* 0.05), compared to miR-control-transfected cells.

**Fig. 6:**
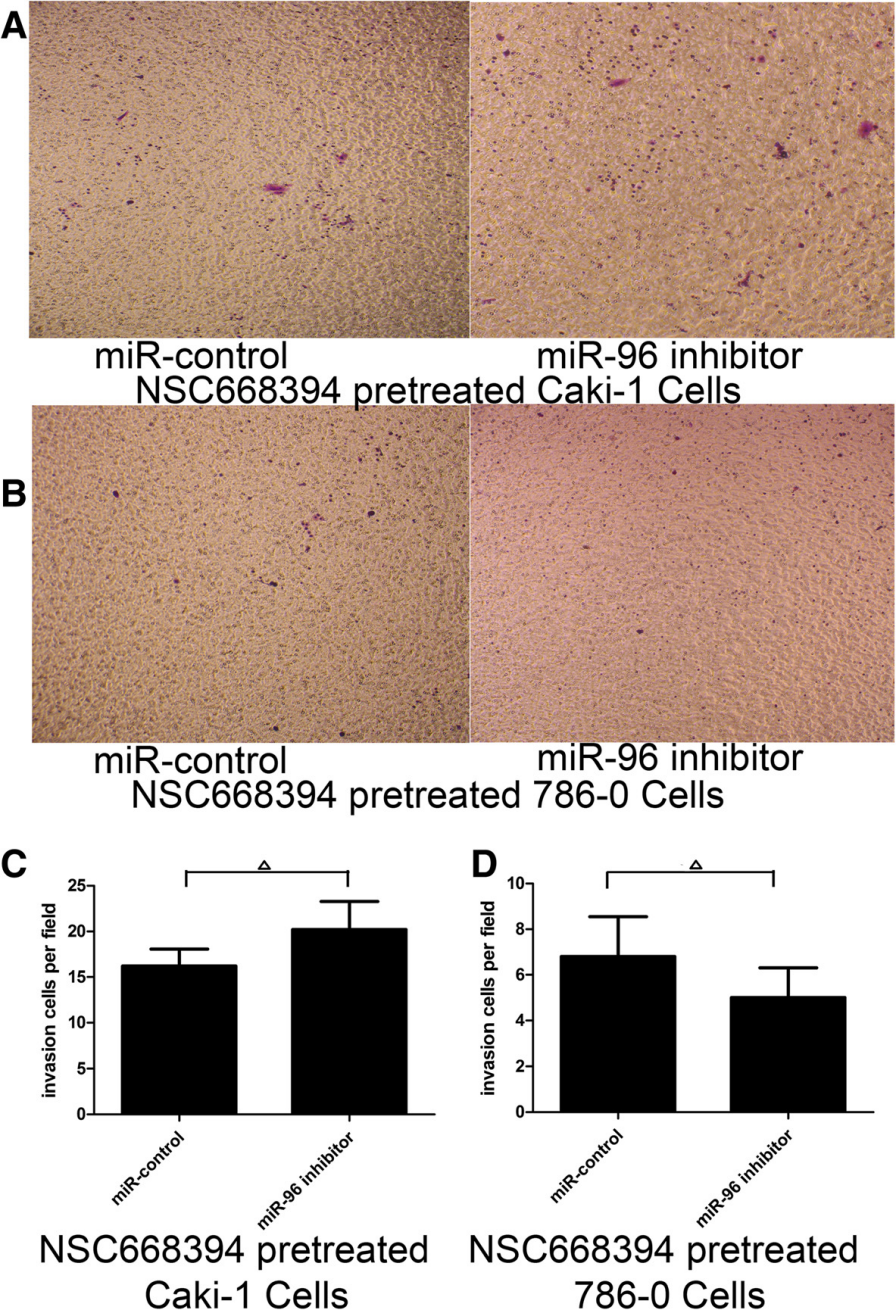
Invasive ability detected by Transwell assay of Ezrin inhibitor-pretreated Caki-1 (**a**) and 786-O (**b**) cells transfected with miR-96 inhibitor or miR-control. RCC cells were pretreated with 10 μM of Ezrin inhibitor NSC668394 for 30 min, followed by transfection with miR-control or miR-96 inhibitor for 24 h. No significant change in mean invading cells per field of NSC668394-pretreated Caki-1 (**c**) and 786-O (D) cells was observed after transfection with miR-96 inhibitor or miR-control (^∆^p>0.05)

As shown in Fig. 6, there was no significant change in the number of NSC668394-pretreated Caki-1 and 786-O cells invading through the Matrigel after transfection with miR-96 inhibitor or miR-control.

### Association of miR-96 levels with Ezrin expression and metastasis in human renal cancer specimens

**Fig. 7:**
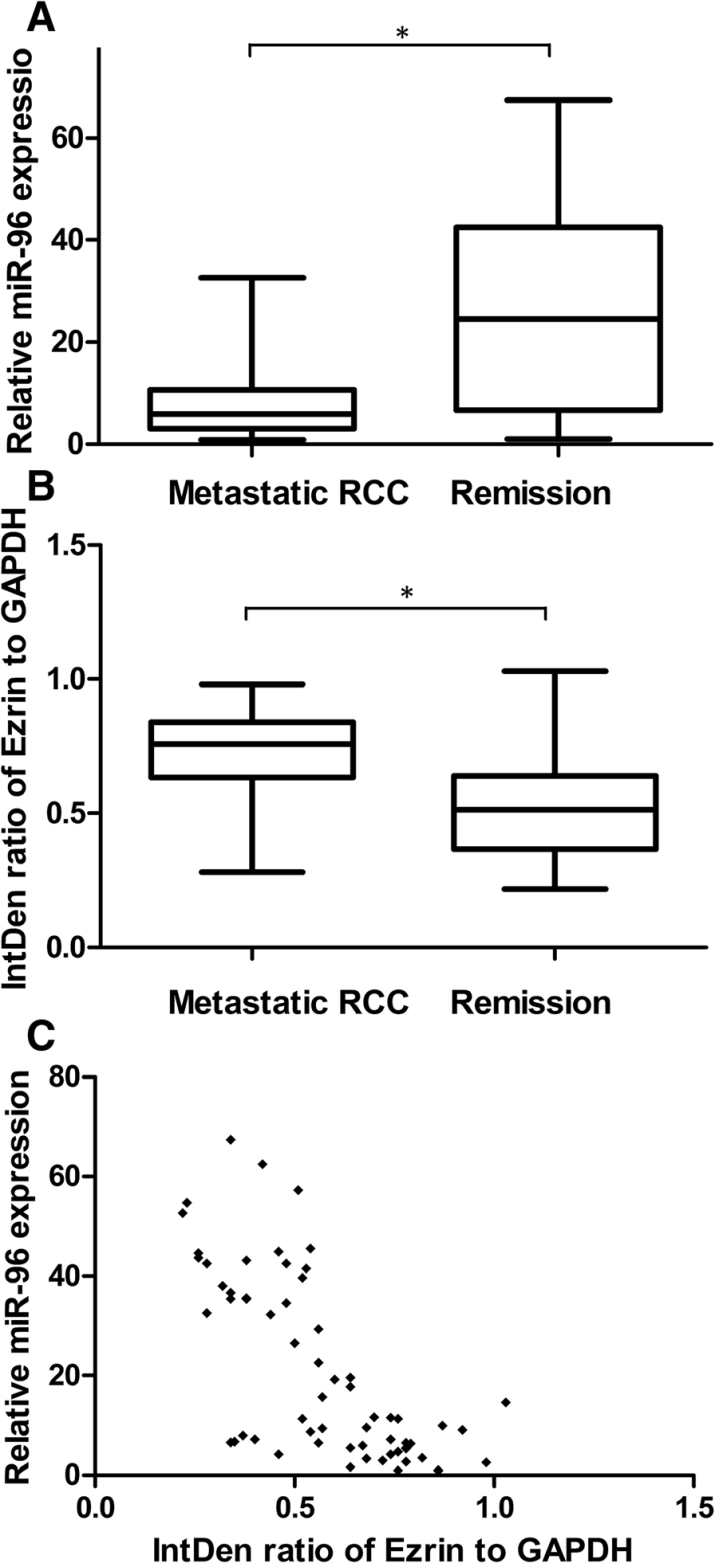
Expression of Ezrin and miR-96 in metastatic and non- metastatic renal cancer patients. **a** miR-96 was lower in patients who developed metastasis than in those who remained in remission. miR-96 expression was detected by quantitative RT-PCR (* *p* < 0.05). **b** Ezrin was higher in patients who developed metastasis than in those who remained in remission. Ezrin was detected by western blotting and normalized to GAPDH. IntDen ratio of Ezrin to GAPDH represents the integrated density ratio as quantified with Image J software. **c** Pearson correlation analysis of the association between miR-96 and Ezrin in renal cancer patients showed r = −0.69 (* *p* < 0.05)

To further investigate the relationship among levels of miR-96, Ezrin and metastasis in a clinical setting, 63 human localized clear cell RCC specimens were analyzed. Using 2010 AJCC TNM staging, there were 39 stage 1 and 24 stage 2 patients. Furhman grades of the 63 RCC were 9 of grade Furhman1, 27 of Furhman 2, 19 of Furhman 3 and 8 of Furhman 4. Seventeen patients developed metastasis, whereas the remaining 46 maintained remission during a median follow-up period of 37 months. The expression levels of miR-96 and Ezrin were compared between patients who developed metastasis and those in remission. As shown in Fig. 7, metastatic patients showed lower levels of miR-96 (*P* < 0.05) and higher levels of Ezrin expression (*P* < 0.05). Pearson correlation analysis of the association between miR-96 and Ezrin in these 63 patients showed a correlation coefficient of–0.69 (*P* < 0.05).

## Discussion

The present study showed that miR-96 expression was negatively correlated with the metastatic potential of RCC cells, and overexpression of miR-96 with synthetic miR-96 oligonucleotides decreased the invasive potential of RCC cells. These results suggest that miR-96 may function as a tumor-suppressing miRNA in renal cancer. To the best of our knowledge, this is the first study investigating the relationship between miR-96 and invasion in renal cancer cells. The impact of miR-96 on tumors has been reported previously in other types of cancer. Yu *et al.* showed that miR-96 directly targets the KRAS oncogene and functions as a tumor-suppressor miRNA in pancreatic cancer cells, where it decreased cancer cell invasion and migration and slowed tumor growth by downregulating KRAS [11]. Vishuamitra *et al.* found that transfection of cell lines with miR-96 decreased proliferation, colony formation, and migration of anaplastic lymphoma kinase-expressing cancer cells [26]. miR-96 was also shown to function as a tumor-promoting miRNA by increasing the invasive ability of tumors in hepatocellular carcinoma cells [7], human bladder urothelial carcinomas [12], non-small cell lung cancer [10], and breast cancer [9]. Xu *et al.* showed that miR-96 expression was positively correlated with liver metastasis in colorectal cancer [27]. Based on the above studies, it appears that miR-96 may work either as a tumor suppressor or promoter, depending on the tumor cell type.

Previous studies have compared miRNA profile changes between metastatic RCC and primary tumors by miRNA microarray [28–31]. However, in those studies, miR-96 did not show significant differences in expression between RCC metastases and primary tumors, which differs from our present results. One possible explanation for this discrepancy is that in the present study, we compared the expression of miR-96 between primary RCC samples of 17 patients who developed metastasis and 46 patients who maintained remission for 37 months, whereas in the studies by Heinzelmann *et al.* [31], Jung *et al.* [28] and White *et al.* [29], miRNA expression was compared between matched metastatic RCC and primary RCC samples in the same patients. Wu *et al.* compared miRNA expression between T1 and T4 stage RCC samples [30]. Another possible reason for the differences in the results of these various studies is the different sensitivity of miRNA microarray vs. qRT-PCR for the detection of changes in miRNA expression, as well as differences in the definition of what constitutes a significant difference in miRNA expression among the studies. Even the studies by Heinzelmann *et al.* [31], Jung *et al.* [28] and White *et al.* [29], which compared miRNA expression between matched metastatic and primary RCC samples, showed different miRNA profiles in the same types of samples. In another report by Heinzelmann *et al.* [32], the miRNA profiles of patients who developed metastasis were compared to those of patients who maintained remission during a specific follow-up period, similar to the present study; however, differential expression of miR-96 was not detected. It is possible that the sample size of our present study and that of Heinzelmann *et al.* may not have been great enough to eliminate the impact of sampling bias.

There were several limitations in our present study. Our results showed a negative correlation between Ezrin and miR-96; however, the precise mechanism by which miR-96 affects Ezrin expression is not clear and warrants further investigation. Furthermore, additional pathways may be involved in miR-96 regulation of invasion in RCC. *In vivo* studies will be required to verify that the progression of RCC can be inhibited by miR-96.

Patients diagnosed at the metastatic stage of RCC have a relatively poor prognosis, with a five year survival rate of only 11.9 % in 2001 in North America [33]. Even in the era of targeted therapy, the median survival for clear cell metastatic RCC has only improved from 11 to 14 months [34]. Further investigation of the potential suppression of RCC invasion by miR-96 and its effect on the downregulation of Ezrin expression may offer novel therapeutic targets to prevent invasion and metastasis of RCC.

## Conclusions

In conclusion, it was found that expression of miR-96 was negatively correlated with the metastatic ability of RCC, and that downregulation of miR-96 could suppress the invasion of renal cancer cell via downregulation of Ezrin expression.

## Abbreviations

RCC: Renal cell carcinoma
miRNAs: MicroRNAs
qRT-PCR: Quantitative reverse transcription-polymerase chain reaction
